# Klotho: a potential therapeutic target in aging and neurodegeneration beyond chronic kidney disease—a comprehensive review from the ERA CKD-MBD working group

**DOI:** 10.1093/ckj/sfad276

**Published:** 2023-11-03

**Authors:** Mehmet Kanbay, Sidar Copur, Lasin Ozbek, Ali Mutlu, Daniel Cejka, Paola Ciceri, Mario Cozzolino, Mathias Loberg Haarhaus

**Affiliations:** Department of Medicine, Nephrology, Koc University School of Medicine, Istanbul, Turkey; Department of Medicine, Koc University School of Medicine, Istanbul, Turkey; Department of Medicine, Koc University School of Medicine, Istanbul, Turkey; Department of Medicine, Koc University School of Medicine, Istanbul, Turkey; Department of Medicine III – Nephrology, Hypertension, Transplantation, Rheumatology, Geriatrics, Ordensklinikum Linz – Elisabethinen Hospital, Linz, Austria; Department of Health Sciences, Renal Division, University of Milan, Milan, Italy; Department of Health Sciences, Renal Division, University of Milan, Milan, Italy; Division of Renal Medicine, Department of Clinical Science, Intervention and Technology, Karolinska University Hospital, Karolinska Institutet, Stockholm, Sweden

**Keywords:** aging, chronic kidney disease, Klotho, neurodegeneration

## Abstract

Klotho, a multifunctional protein, acts as a co-receptor in fibroblast growth factor 23 and exerts its impact through various molecular pathways, including Wnt, hypoxia-inducible factor and insulin-like growth factor 1 pathways. The physiological significance of Klotho is the regulation of vitamin D and phosphate metabolism as well as serving as a vital component in aging and neurodegeneration. The role of Klotho in aging and neurodegeneration in particular has gained considerable attention. In this narrative review we highlight several key insights into the molecular basis and physiological function of Klotho and synthesize current research on the role of Klotho in neurodegeneration and aging. Klotho deficiency was associated with cognitive impairment, reduced growth, diminished longevity and the development of age-related diseases *in vivo*. Serum Klotho levels showed a decline in individuals with advanced age and those affected by chronic kidney disease, establishing its potential diagnostic significance. Additionally, multiple medications have been demonstrated to influence Klotho levels. Therefore, this comprehensive review suggests that Klotho could open the door to novel interventions aimed at addressing the challenges of aging and neurodegenerative disorders.

## INTRODUCTION

Aging and neurodegenerative disorders are complex medical conditions with poorly understood underlying pathophysiology that may affect virtually all tissues and organs. A limited number of genes and their transcripts have been associated with either premature aging, as seen in progeria, or with longevity [1, 2]. The α-Klotho protein, first identified in mice studies in 1997 [3], primarily functions as a co-receptor for fibroblast growth factor 23 (FGF23) in the kidneys and parathyroid gland and therefore has a crucial role in phosphate homeostasis, vitamin D metabolism and vascular calcification [4]. In addition, α-Klotho has been identified in a wide variety of tissues, consistent with its role in the aging process, including endocrine organs, arteries and reproductive, epithelial and neuronal tissue [5, 6]. β-Klotho is primarily expressed in the liver, adipose tissue and kidneys and plays a role in lipid and energy metabolism by acting as co-receptor for FGF15 and FGF21 [7, 8], while the γ-Klotho isoform is expressed in the brown adipose tissue, skin and kidneys with as yet poorly defined roles and by acting as co-receptor for FGF receptor 1b (FGFR1b), FGFR1c, FGFR2c and FGFR4 [4, 9, 10] and are not discussed in the current review. Nevertheless, preclinical studies conducted on Klotho knockout mice have revealed that Klotho deficiency is associated with impaired cognition, shorter lifespan, cardiac hypertrophy, vascular calcification, multi-organ atrophy and fibrosis and growth retardation [3, 11]. Moreover, overexpression of the Klotho gene has been shown to lengthen lifespan in mice [12], which raises the potential question of whether Klotho may be utilized as a target to control or reverse aging and/or neurodegeneration. One drawback of such models is the lack of distinction between soluble and transmembrane Klotho molecules, which may potentially have distinct physiological roles in the human body. Whether such physiological and pathological outcomes of Klotho deficiency and/or overexpression may simply be attributable to the role of Klotho on phosphate metabolism is unclear and should be evaluated with caution with the discovery of multiple phosphate metabolism–independent functions of Klotho protein. In this narrative review, our aim is to evaluate the potential pathophysiological and therapeutic role of Klotho protein in aging and neurodegenerative conditions.

### The molecular basis

Klotho is a single-pass 130-kD transmembrane protein consisting of a short cytoplasmic tail, a transmembrane domain and two extracellular domains referred to as Kl1 and Kl2 and primarily expressed in kidneys, parathyroid glands and choroid plexus [13]. Membrane-bound Klotho functions as a co-receptor for FGF23 and FGFRs, while cleavage of the extracellular domains of Klotho protein leads to the formation of soluble Klotho [14]. The extracellular domain of Klotho is released into the blood circulation through cleavage by a disintegrin and metalloproteinase domain-containing protein 10 (ADAM10) and ADAM17, which is stimulated by insulin and inhibited by metalloproteinase inhibitors [15]. It has been demonstrated that the kidney has a dual role in Klotho homeostasis, as it has been shown to produce and release Klotho into the circulation while also clearing Klotho from the blood into the urinary lumen [13]. Soluble Klotho is the primary form of Klotho present in urine via tubular transcytosis, not glomerular filtration [13], and acts as a paracrine and endocrine signal in many organ systems [16].

#### Klotho gene

The *Klotho* gene is located at chromosome 5 in humans between the *PDS5B* and *STARD13* genes, which is homologous to the rat and mouse genomes [17]. The Klotho gene includes five exons and four introns [18]. The transcription of Klotho is downregulated in response to pro-inflammatory conditions, angiotensin II, nuclear factor κ light chain enhancer of activated B cells (NF-κB) signalling, matrix metalloproteinases and certain conditions such as dehydrated states and diabetic nephropathy [19–21]. On the other hand, vitamin D, the peroxisome proliferator-activated receptor γ (PPAR-γ) signalling pathway and multiple medications such as losartan, statins, fosinopril, erythropoietin and rapamycin upregulate *Klotho* gene expression [17, 22–24].

#### Physiological functions

The Klotho protein has multiple physiological functions in various organs systems through multiple signalling pathways.

#### Phosphate homeostasis

FGF23, a bone-derived hormone, is a phosphaturic hormone with gain-of-function mutations leading to resistance towards degradation by PHEX (phosphate regulating endopeptidase homolog, X-linked), leading to autosomal dominant hypophosphataemic rickets. FGF23 has weak affinity towards FGFRs in proximal tubules, while Klotho protein is crucial in the maintenance of such a link. The N-terminal region of FGF23 binds to FGFRs and the C-terminal region interacts with Klotho protein, while completion of such a complex leads to inhibition of phosphate reabsorption through proximal tubules due to downregulation of NaPi-2a and NaPi-2c and inhibition of active vitamin D generation due to downregulation of enzyme 1-alpha hydroxylase [25, 26]. Other physiological actions of FGF23 on phosphate metabolism include the upregulation of enzyme 24-hydroxylase, which is involved in the inactivation of active vitamin D and attenuation of parathyroid hormone release [27, 28].

#### Wnt signalling pathway

The Wnt/β-catenin signalling pathway is involved in cell cycle arrest at the G2/M phase and upregulation of pro-fibrotic cytokines and molecules [29]. Also, the Wnt/β-catenin signalling pathway is a universal pathway involved in embryogenesis via axis development, cell proliferation and cell migration [29]. Klotho has been shown to inhibit the Wnt signalling pathway via binding to multiple natural Wnt ligands [30]. Klotho is also involved in the regulation of intracellular calcium homeostasis [31], which may lead to inducement of μ-calpain, a calcium-dependent protease, thus leading to degradation of β-catenin [32]. Moreover, a decrease in the levels of Klotho in chronic kidney disease (CKD) patients may contribute to the development of kidney injury and fibrosis due to loss of suppression on the Wnt/β-catenin signalling pathway [32]. Additionally, augmented Wnt signalling has been linked to stem and progenitor cell dysfunction and cellular senescence. Therefore, Klotho-mediated downregulation of the Wnt signalling pathway may reverse such a condition and improve tissue regeneration [30].

#### Insulin-like growth factor 1 (IGF-1) signalling pathway

Multiple studies have demonstrated that Klotho suppresses the downstream signalling pathways of IGF or insulin receptor substrate (IRS) without direct interaction [33, 34]. One possible explanation is through the interactions over forkhead box proteins (FOXOs) in which activation of the IGF-1 signalling pathway results in upregulation of the PI3K/Akt pathway and phosphorylation of FOXO1/3a/4 proteins. Such phosphorylation prevents the nuclear translocation of FOXOs and thereby interferes with their transcription activity, including glucose-6-phosphatase activity involved in gluconeogenesis in the liver [35]. Also, FOXO1 is involved in the downregulation of adipogenesis via binding to the promoter region of PPARγ while Klotho protein leads to contradictory action via binding to the same region [36]. Another anti-aging property of Klotho protein is the inhibition of the IGF-1 signalling-mediated increase in reactive oxygen species, leading to downregulation of oxidative stress [34].

#### Hypoxia-inducible factor (HIF) pathway

HIF [37] is a protein complex essential for controlling vascularization and homeostasis. It promotes vascularization in hypoxic areas and acts as a transcription factor for many target genes in response to low oxygen levels [38]. It has been indicated that the transcription level of HIF1α protein is negatively regulated by Klotho [39]. Moreover, exogenous supplementation of Klotho can reduce the HIF level [40]. Klotho deficiency leads to an increase in erythropoietin (EPO) production through HIF activation. However, this increase in EPO production does not lead to an improvement in haematopoiesis because it is observed that haematopoietic stem cells in bone marrow of klotho-deficient mice are decreased [41, 42]. Shedding of soluble Klotho by the kidneys is induced by hypoxia [43], thus indicating a negative feedback loop as part of the complex interaction between tissue oxygenation, HIF1α, EPO, FGF23 and Klotho.

#### Enzymatic role

The two internal repeats of the extracellular domain of Klotho protein, namely KL1 and KL2, have structural and amino acid sequence homology to family 1 glycosidase enzymes that hydrolyse β-glycosidic bonds in glycoproteins and glycolipids [44]. Nevertheless, such β-glycosidic activity has not been detected in *in vitro* studies [45]. On the other hand, Klotho protein exerts β-glucuronidase and sialidase activity in *in vitro* models [31]. Such activity is involved in the regulation of transcellular transport of phosphate in renal proximal tubules via proteolytic degradation of NaPi-2a transporter via β-glucuronidase activity of Klotho, leading to phosphaturia [44, 46]. Nevertheless, there is a clear need for future studies for a better understanding of the enzymatic role of Klotho protein in human physiology.

#### Others

α-Klotho and soluble Klotho proteins are involved in multiple other physiological events, including the upregulation of antioxidant defence mechanisms, synthesis of proteins involved in the cell cycle such as p15 and p21, regulation of senescence and apoptosis via p53 or transforming growth factor β (TGF-β) signalling pathway [47, 48].

### Klotho as an anti-aging target

The serum levels of Klotho have been shown to decrease with certain diseases, including CKD, neurodegenerative conditions, diabetes mellitus and aging. A recent cross-sectional cohort study including 346 healthy adult participants 18–85 years of age demonstrated that senior adults (ages 55–85 years) have the lowest serum α-Klotho levels (*P* < .01) and serum Klotho levels have negative correlation with age (*P* < .001) [49]. Moreover, Klotho-deficient mice have illustrated higher rates of vascular calcification, cardiac hypertrophy, cognitive impairment, hypercalcaemia, hyperphosphataemia, multi-organ atrophy and osteopenia [3, 50]. A national cohort study in the USA involving 10 069 adult participants ages 40–79 years with serum Klotho levels measured via enzyme-linked immunosorbent assay revealed that participants with low serum levels of Klotho (<666 pg/ml) have 31% higher risk of all-cause mortality compared with participants with higher serum levels of Klotho (>985 pg/ml) {hazard ratio 1.31 [95% confidence interval (CI) 1.00–1.71], *P* = .05} during a median follow-up period of 58 months. Such a statistically significant association persists for cardiovascular disease–related mortality and malignancy-related mortality [51]. Such a negative association between serum Klotho levels and all-cause mortality has also been established in another observational cohort study conducted in Italy, involving >804 adult participants ≥65 years of age [52]. Moreover, a meta-analysis of six cohort studies, including adult patients with CKD, revealed similar outcomes of higher risk for all-cause mortality among patients with lower serum Klotho levels [relative risk 1.88 (95% CI 1.29–2.74), *P* < .05] [53]. Furthermore, overexpression of the *Klotho* gene in transgenic mice has been shown to reverse or delay aging in preclinical studies, however, the therapeutic applicability of recombinant Klotho in human subjects is unknown [12].

Increasing Klotho levels emerges as a promising strategy in CKD. Experiments in rodents via viral delivery of Klotho cDNA or stimulating endogenous production through various means have shown promising results [54]. These methods demonstrate improvements in kidney function and protection from related complications [54]. Existing drugs such as paricalcitol and sevelamer carbonate also show potential in elevating Klotho levels [16]. However, clinical application faces challenges, with safety concerns and a lack of knowledge regarding upregulation and degradation mechanisms. Administering exogenous Klotho protein has demonstrated effective results in animal models [55], offering both restoration of levels and promotion of endogenous production, which is crucial in kidney failure scenarios. Despite success in animal trials, implementing Klotho replacement therapy in humans encounters significant obstacles [16]. A study conducted by Zhong *et al.* [56] investigated soluble Klotho (s-Klotho) derived from two distinct mammalian expression systems and found notable differences in their *in vitro* activities, *in vivo* pharmacokinetic properties and post-translational modifications (PTMs). A mutant s-Klotho with enhanced glycosidase functions exhibited reduced FGF23 co-receptor activity, suggesting structural divergence between the two well-established functions of s-Klotho. These findings offer valuable insights into the structure–function relationship of s-Klotho and its PTM biology, providing guidance for future therapeutic protein engineering of s-Klotho [56]. Additionally, fragment-based drug discovery methods could potentially overcome obstacles such as screening problems, early degradation resulting in a short half-life, low potency and challenges in crossing barriers. This approach may be particularly beneficial for developing small peptides of Klotho or other molecules with similar efficacy.

Multiple currently available drugs have been shown to upregulate serum levels of Klotho in human subjects.

#### Sodium–glucose co-transporter 2 (SGLT2) inhibitors

SGLT2 inhibitors constitute a new class of oral antidiabetic agents that improve glycaemic control by increasing renal sodium and glucose excretion. In addition to, and independent of, the effect on blood glucose, SGLT2 inhibitors have cardio- and renoprotective effects and can slow CKD progression [57]. Experimental studies have demonstrated that SGLT2 inhibitors prevent the downregulation of Klotho by inflammatory cytokines and high glucose in renal tubular cells, while they improved serum and urine Klotho levels and decreased urinary inflammatory markers and albuminuria in patients with early diabetic nephropathy [58]. In an animal model of unilateral ureteric obstruction, the SGLT2 inhibitor empagliflozin partially inhibited kidney function decline and the activation of inflammatory and profibrotic pathways [59]. Despite these promising clinical and experimental results, further studies are required to elucidate the role of Klotho in the cardio- and renoprotective effects of SGLT2 inhibitors.

#### Renin–angiotensin–aldosterone system (RAAS) inhibitors

RAAS blockers are commonly prescribed medications used in the management of hypertension, congestive heart failure, diabetic nephropathy, CKD and hyperaldosteronism. Losartan and valsartan have been shown to lead to an upregulation in serum Klotho levels in preclinical and clinical studies, however, the clinical importance of such an observation is less well understood [60–63]. The efficiency of valsartan and fluvastatin therapy separately or in combination in terms of anti-aging properties has been evaluated in a study including 130 healthy adult male participants. Participants received either fluvastatin 10 mg/day alone, valsartan 20 mg/day alone, fluvastatin 10 mg/day and valsartan 20 mg/day in combination or placebo for 30 days. Blood samples were obtained prior to therapy, after 30 days of therapy and 5 months after the discontinuation of therapy, while expression levels of six genes (*SIRT1, PRKAA, KLOTHO, NFE2L2, mTOR* and *NF-κB*) were analysed. The combination therapy leads to a 1.8-fold increase in *SIRT1* (*P* < .0001), a 1.5-fold increase in *PRKAA* (*P* = .262) and a 1.7-fold increase in *KLOTHO* (*P* < .0001) levels while either therapy alone only leads to a slight increase in *SIRT1* expression. Such a pattern of gene expression disappeared following the discontinuation of therapy [64]. This study is significant by demonstrating alterations in gene expression levels with commonly utilized medications, however, whether such alterations have clinical significance in terms of mortality or morbidity is unclear. Such an association has been validated in a randomized controlled clinical trial conducted in 76 patients with type 2 diabetes mellitus and diabetic kidney disease (DKD) in which participants were randomized to receive either valsartan/hydrochlorothiazide (*n* = 37) or amlodipine therapy (*n* = 39). Participants receiving valsartan/hydrochlorothiazide therapy had a statistically significant increase in serum Klotho levels (from 432.7 ± 179 to 506.4 ± 226.8 pg/ml; *P* = .01) even after comparison with the amlodipine group (*P* = .03) [60]. Furthermore, a similar pattern of elevation at serum Klotho levels in patients with DKD has been demonstrated with losartan therapy as well [62].

On the other hand, there is no clinical trial, to the best of our knowledge, conducted on human subjects regarding the effects of spironolactone or epleronone, aldosterone receptor antagonists, on serum Klotho levels. A preclinical trial conducted on mice and human embryonic kidney cells demonstrated that spironolactone therapy leads to upregulation of Klotho gene expression [65]. Nevertheless, there is a clear need for confirmatory studies in human participants for a better understanding of this issue.

#### Statins

Statins, 3-hydroxy-3-methyl-glutaryl-coenzyme A (HMG-CoA) reductase inhibitors, are widely used medications in the management of atherosclerosis, hyperlipidaemia, ischaemic cardiovascular or cerebrovascular diseases and peripheral artery diseases. Atorvastatin, pitavastatin and simvastatin have been shown to upregulate Klotho expression in preclinical studies [24, 66, 67]. An animal model of cognitive decline induced via intracerebroventricular streptozotocin administration has been utilized to evaluate the response to statin therapy in terms of cognitive functions. Klotho expression decreased significantly following the administration of streptozotocin compared with control models. Spatial performance was improved in animal models treated with simvastatin for 3 weeks, which may be attributable to the upregulation of Klotho and manganese superoxide dismutase [68].

#### Mammalian target of rapamycin (mTOR) inhibitors

The mTOR inhibitors are widely used immunosuppressive medications utilized in autoimmune disorders and transplantation. Serum Klotho levels were evaluated in an observational study including 36 kidney transplant recipients either taking everolimus or not in which serum Klotho levels were measured before and 1 year after kidney transplantation. Serum Klotho levels were higher 1 year after kidney transplantation as expected with the improvement in renal function (369.3 versus 211.8 pg/ml). Moreover, serum Klotho levels have been found to be higher in patients receiving everolimus therapy compared with others, in a statistically significant manner (536.7 versus 332.4 pg/ml) [69]. Another study conducted on 100 kidney transplant recipients, 50 treated with rapamycin and 50 treated with calcineurin inhibitors, showed similar findings, while elevated Klotho expression has been linked to the activation of mTORC2 via the phosphorylation of AKT [70].

#### Others

PPARγ agonists such as pioglitazone and rosiglitazone and vitamin D supplementations have been shown to upregulate Klotho expression in experimental animal models. Nevertheless, their applicability to human subjects is doubtful [71–75]. Multiple preclinical therapeutic modalities primarily targeting Klotho expression or functions are present in the literature.

#### γ-Aminobutyric acid receptor (GABA) agonists

The use of GABA agonists is a novel therapeutic approach currently in preclinical studies. Administration of GABA has been shown to increase the levels of circulating Klotho and Klotho levels at pancreatic islet of Langerhans among streptozotocin-induced diabetic mice subjects, which may be diminished in Klotho-knockdown subjects. However, infusion of soluble Klotho may reverse such conditions, thus indicating the pathophysiological importance of Klotho in the underlying mechanism of both type 1 diabetes mellitus and GABA-induced improvement [76]. In another study conducted on immunodeficient streptozotocin-induced diabetic mice models with human pancreatic β cell transplantation, the superiority of GABA + dipeptidyl peptidase (DPP)-IV inhibitor, namely sitagliptin, was revealed [77]. A preclinical study conducted on human islet cells incubated under cytokine mixture for 24 hours demonstrated that cytokine-induced apoptosis is significantly reduced by either GABA or glucagon-like peptide-1 analogue, namely exendin-4, therapy. However, the combination therapy has led to superior outcomes via upregulation of Klotho and sirtuin 1 expression and activation of the AKT signalling pathway [78].

#### Recombinant protein and gene therapy

Gene therapy and the use of recombinant proteins in the management of certain conditions are novel areas of medical research, while considerable efforts have been made regarding such therapeutic alternatives linked to Klotho over the last decade. Supplementation with recombinant Klotho has been shown to protect against kidney fibrosis caused by hypoxia or other insults via inhibiting TGF-β signalling in cell lines and animal models [79–81]. Also, recombinant Klotho therapy has been shown to exert protective actions on myocardial ischaemia–reperfusion injury, insulitis in diabetic subjects and hypertension in various models [40, 82, 83]. Moreover, Klotho-derived peptide 6 (KP6) mimics the function of the anti-aging protein Klotho and shows promise in treating DKD. KP6 reversed proteinuria, reduced kidney damage and inhibited β-catenin activation, suggesting a potential therapeutic strategy for DKD treatment [84]. Furthermore, gene therapies focusing on Klotho are being evaluated for therapeutic alternatives for conditions such as diabetes mellitus and neurodegenerative diseases such as epilepsy and Alzheimer's disease [85–87]. Nevertheless, such therapeutic alternatives are far from any clinical use and require multiple large-scale studies evaluating their efficiency and safety in human subjects.

### Diet and nutrition

**Figure 1:**
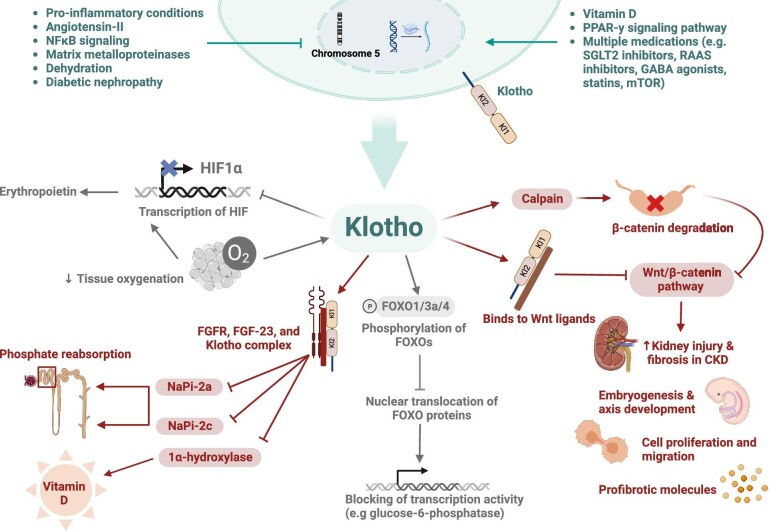
Summary of the molecular basis and physiological impacts of Klotho. Factors that negatively affect Klotho production are shown in the upper left part of the figure and those that have positive impacts are shown in the upper right. The molecular pathways affected by Klotho, including IGF-1, Wnt, HIF [36] and phosphate homeostasis, are shown. The respective physiological alterations are depicted. Blunt and conventional arrows indicate inhibition and activation, respectively.

The serum level of Klotho is also affected by food and diet types. To decipher the role of diet in the serum level of Klotho is crucial to its relationship with aging and related diseases. A cross-sectional study based on 2007–2016 data with 7906 participants in the USA indicated that the serum Klotho level significantly increases [β coefficient 9.41 (95% CI 6.08–12.74), *P* < .001; 9.41 pg/ml increase per 1 point in the Mediterranean Diet Score) in people who adhere to a Mediterranean diet, probably due to a synergistic effect of food combinations, while in people who prefer low-fat and low-carbohydrate diets, non-significant associations were seen [88]. Additionally, a cross-sectional study with 8456 participants ages 40–79 years in the USA demonstrated a positive correlation between a Healthy Eating Index 2015 score and the serum Klotho level [β 0.74 (95% CI 0.21–1.27), *P* = .0067] [89]. Moreover, another study showed that Klotho is a significant mediator in the enhancement of kidney function by a healthy diet (Healthy Eating Index 2015 score) [β 0.05 (95% CI 0.02–0.08), *P* < .001] [90]. According to a cross-sectional study based on data from 2015–2016 with 2637 participants, 40–79 years old, in the USA, a high-fibre diet in men increases serum Klotho level [91], while a similar study with 11 282 participants (2007–2016 data from participants 40–79 years old in the USA) showed that a high-fibre diet in overweight, obese and elderly participants has a more robust association with serum Klotho levels [92]. Although the benefit of a healthy and high-fibre diet on Klotho levels has been shown in multiple studies, it is crucial to note that the connection between serum Klotho and diet is not entirely understood. More studies are required to demonstrate a definite causal relationship between eating habits and Klotho levels. Furthermore, genetics, lifestyle variables and other environmental influences all have a role in klotho levels and overall health. Fig. 1 demonstrates the summary of negative and positive factors affecting Klotho production as well as the signalling pathways with which Klotho interacts to exert its physiological impacts.

### Klotho in aging

Aging can be defined as an irreversible process that leads to the gradual deterioration of tissue and organ function, eventually resulting in death. It is characterized by molecular and cellular changes such as genomic instability, oxidative stress, mitochondrial dysfunction and telomere shortening [93]. The role of Klotho protein in aging has been evaluated in multiple preclinical and clinical studies. Overexpression of Klotho mediated via the control of human elongation factor 1α promoter has been linked to an increase in the lifespan of mice [12]. A meta-analysis regarding the genetic variation of Klotho and its relation to longevity indicated a significant association between healthy aging and the KL-VS variant of the *Klotho* gene [94]. On the other hand, a case–control study showed no significant relation between the KL-VS variant of *Klotho* and longevity [95]. A prospective cohort study conducted in 804 adult participants ≥65 years of age with a 6-year follow-up period demonstrated that low serum Klotho levels (<575 pg/ml) are linked to a higher risk for mortality compared with higher serum Klotho levels (<763 pg/ml) after multivariate adjustments [52]. Another prospective study conducted in 1023 adult participants demonstrated that plasma Klotho levels are inversely correlated with age (*P* < .001) and serum C-reactive protein levels (*P* < .001). Moreover, age-adjusted serum Klotho levels are inversely correlated with the risk of cardiovascular disease (*P* < .001). Additionally, after adjustment for multiple cardiovascular disease–related variables (age, gender, smoking, lipid profile, diabetes mellitus, systolic blood pressure), log plasma Klotho levels were associated with cardiovascular disease risk [odds ratio per 1 standard deviation increase = 0.85 (95% CI 0.72–0.99)] [96]. Other cohort studies have illustrated a statistically significant inverse correlation between serum Klotho levels and cognitive decline or physical performance [97, 98].

Furthermore, several studies have shown that serum Klotho levels are inversely correlated with age. In other words, younger people tend to have higher plasma Klotho levels [99, 100]. Also, deficiency of Klotho contributes to hypertension, arterial stiffening, endothelial dysfunction and many other age-associated disorders [4]. It has been shown that expression impairments of the *Klotho* gene in mice produce a bundle of symptoms similar to aging in humans, such as decreased lifetime, skeletal and dermal problems, infertility and arterial stiffening [3].

Nevertheless, one significant concern and confounding factor in such research is the clear link between CKD and aging irrespective of serum Klotho levels [101, 102]. Multiple pathophysiological mechanisms, including uraemic toxin–mediated alterations, traditional cardiovascular disease–related neurodegeneration, polypharmacy, anaemia and dialysis-related factors are involved in this complex pathway [102]. Therefore it is important not to overlook such confounding factors in the discussion and evaluation of the role of Klotho in aging and neurodegenerative conditions.

### Klotho in neurodegenerative disorders

**Figure 2:**
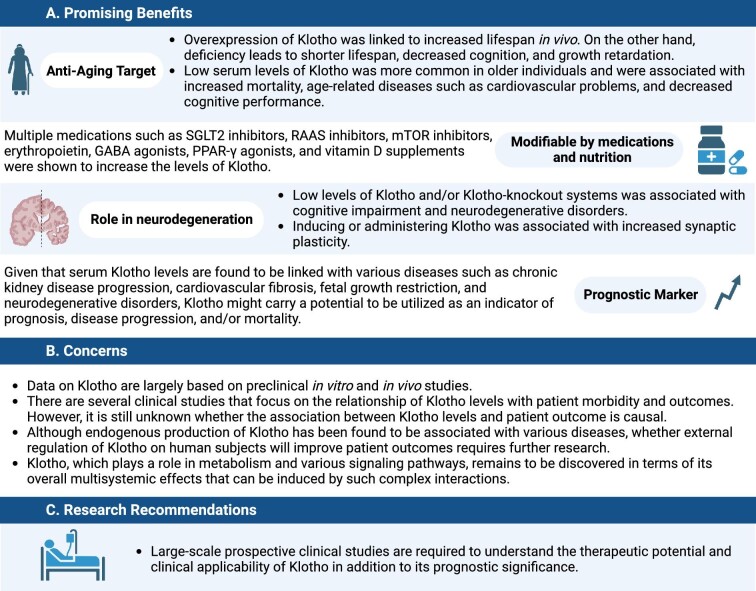
Infographic summary of the promising aspects, concerns and research recommendations of Klotho.

The highest level of Klotho production in the brain occurs at the choroid plexus, while lower production occurs in the hippocampus, substantia nigra, medulla and cerebral/cerebellar cortex [103]. Klotho expression is seen in both neurons and oligodendrocytes, while the production starts *in utero* and diminishes with age. Mean cerebrospinal fluid Klotho levels are significantly lower in older adults compared with younger individuals (*P* = .005) and in patients with Alzheimer's disease (*P* = .02) [104]. An observational study conducted in 103 elderly patients in Spain revealed that lower serum Klotho levels in adults are associated with higher frailty and more likely to experience falls in the following 6 months [105]. The physiological functions of Klotho in the central nervous system have initially been illustrated via studies conducted on Klotho-knockout animal subjects and include diminished axonal transport, formation of a myelin sheath and maturation of oligodendrocytes, a decrease in the proliferation of hippocampal neural progenitor cells and cognitive impairment [106, 107]. Additional physiological functions have been identified via studies including administration of recombinant Klotho or epigenetic studies inducing the overexpression of Klotho protein. Incubation of hippocampal neuronal cell cultures with soluble Klotho leads to a decrease in oxidative stress via upregulation of superoxide dismutase activity and pro-inflammatory cytokines such as interleukin-1, tumour necrosis factor-α and NF-κB and causes protection against amyloid-β or glutamate-induced oxidative toxicity [108, 109]. Administration of soluble Klotho peripherally to mice subjects has led to enhancement of synaptic plasticity and glutamate receptor signalling, while similar findings have been recorded when secreted Klotho is overexpressed in mice models [110, 111]. Furthermore, overexpression of the full-length *Klotho* gene results in a decrease in oxidative and endoplasmic reticulum stress, inhibition of NLRP3 inflammasomes, improvement in synaptic plasticity and amyloid-β clearance mechanisms, protection of dopaminergic and hippocampal neurons and improvement in memory and cognition (Fig. 2) [112–115].

On the other hand, Klotho does not cross the blood–brain barrier (BBB). However, Leon *et al.* [111] demonstrated that administering the alpha Klotho fragment (α-KL-F) peripherally results in an immediate enhancement of cognitive function and heightened neural resilience and plasticity in mice at various life stages. Interestingly, peripherally administered α-KL-F shows its effect by cleavage of the N-methyl-D-aspartate receptor (NMDAR) subunit GluN2B and results in increased NMDAR-dependent synaptic plasticity [111]. Additionally, it has been shown that platelet factor 4 induced by Klotho can cross the BBB and can increase hippocampal synaptic plasticity [116]. Despite the promising discoveries regarding the action and mechanism of Klotho, there are still crucial gaps in the literature, especially how Klotho exerts its effects on memory and neurocognition.

## CONCLUSION

Knowledge regarding the physiological functions of Klotho protein, first identified in 1997, has expanded over the years, despite initial studies primarily focusing on its role in vitamin D metabolism, vascular calcification and phosphate homeostasis, especially among patients with CKD. Preclinical studies have demonstrated potential links between serum Klotho levels and multiple conditions including CKD progression, cardiovascular fibrosis, aging and neurodegenerative disorders. Emerging clinical and experimental insights suggest Klotho deficiency not only as a risk factor, but also a modifiable therapeutic target. Even though there is a clear need for future large-scale human studies in order to develop clinical and therapeutic strategies involving Klotho proteins in humans, this field appears to be promising.
